# Nurse-led home visitation programme to improve health-related quality of life and reduce disability among potentially frail community-dwelling older people in general practice: a theory-based process evaluation

**DOI:** 10.1186/s12875-014-0173-x

**Published:** 2014-10-25

**Authors:** Mandy M N Stijnen, Maria W J Jansen, Inge G P Duimel-Peeters, Hubertus J M Vrijhoef

**Affiliations:** Department of Family Medicine, School for Public Health and Primary Care (CAPHRI), Faculty of Health, Medicine and Life Sciences, Maastricht University, P.O. Box 616, 6200 MD Maastricht, The Netherlands; Public Health Service South-Limburg, P.O. Box 2022, 6160 HA Geleen, The Netherlands; Department of Health Services Research, School for Public Health and Primary Care (CAPHRI), Faculty of Health, Medicine and Life Sciences, Maastricht University, P.O. Box 616, 6200 MD Maastricht, The Netherlands; Department of Patient and Care, Maastricht University Medical Centre, P.O. Box 5800, 6202 MD Maastricht, The Netherlands; Tilburg School of Social and Behavioral Sciences, Scientific Centre for Care and Welfare (TRANZO), Tilburg University, P.O. Box 90153, 5000 LE Tilburg, The Netherlands; Saw Swee Hock School of Public Health, National University of Singapore, MD3, 16 Medical Drive, Singapore, 117597 Republic of Singapore

**Keywords:** Frail elderly, General practice, Geriatric assessment, Home visit, Mixed-methods, Practice nurse, Primary care, Process evaluation, Program implementation

## Abstract

**Background:**

Population ageing fosters new models of care delivery for older people that are increasingly integrated into existing care systems. In the Netherlands, a primary-care based preventive home visitation programme has been developed for potentially frail community-dwelling older people (aged ≥75 years), consisting of a comprehensive geriatric assessment during a home visit by a practice nurse followed by targeted interdisciplinary care and follow-up over time. A theory-based process evaluation was designed to examine (1) the extent to which the home visitation programme was implemented as planned and (2) the extent to which general practices successfully redesigned their care delivery.

**Methods:**

Using a mixed-methods approach, the focus was on fidelity (quality of implementation), dose delivered (completeness), dose received (exposure and satisfaction), reach (participation rate), recruitment, and context. Twenty-four general practices participated, of which 13 implemented the home visitation programme and 11 delivered usual care to older people. Data collection consisted of semi-structured interviews with practice nurses (PNs), general practitioners (GPs), and older people; feedback meetings with PNs; structured registration forms filled-out by PNs; and narrative descriptions of the recruitment procedures and registration of inclusion and drop-outs by members of the research team.

**Results:**

Fidelity of implementation was acceptable, but time constraints and inadequate reach (i.e., the relatively healthy older people participated) negatively influenced complete delivery of protocol elements, such as interdisciplinary cooperation and follow-up of older people over time. The home visitation programme was judged positively by PNs, GPs, and older people. Useful tools were offered to general practices for organising proactive geriatric care.

**Conclusions:**

The home visitation programme did not have major shortcomings in itself, but the delivery offered room for improvement. General practices received useful tools to redesign their care delivery from reactive towards proactive care, but perceived barriers require attention to allow for sustainability of the home visitation programme over time.

## Background

Healthcare professionals worldwide are increasingly called upon to organise and deliver care to a growing number of older people. This stimulated the development of various multifactorial interventions and care models aimed at maintaining independent living and the prevention of disability and other adverse outcomes in community-dwelling older people [1–3]. Especially primary care has been considered ideally suited to address the needs of older people, and more specifically frail older people who are at risk of functional decline and hospitalisation, predominantly due to their patient-oriented focus [4,5]. In several countries with a strongly developed primary healthcare system, such as the UK, Denmark and the Netherlands, interventions comprising comprehensive geriatric assessment (CGA) exist in which the general practitioner (GP) acts as the central care provider [6–8]. However, primary care based models for care for older people also pose challenges to GPs. These relate to difficulties in dealing with multiple and often co-occurring medical conditions, (inter)personal challenges (e.g., communication barriers, time pressure), and the burden of administrative work [9].

There is a growing recognition that the primary care setting, and particularly general practices, have the potential to deliver patient-centred, coherent, and proactive care to older people [4,10]. Therefore, in the south of the Netherlands, the [G]OLD (‘Getting OLD the healthy way’) preventive home visitation programme has been developed aimed at improving health-related quality of life and reducing disability among potentially frail community-dwelling older people in general practice [11]. Care delivery within general practices is redesigned by applying components of the Chronic Care Model (CCM), a model developed to improve chronic illness management in primary care [12,13]. In addition, the Guided Care Model informed the development of the intervention protocol of the [G]OLD home visitation programme. So far, Guided Care seems to be the only evidenced-based model that translated components of the CCM in a stepwise intervention model in an effort to transform care for vulnerable older people with multiple chronic conditions and complex care needs [14]. As a result, the [G]OLD home visitation programme consists of a CGA of older people’s health and well-being during a home visit by the practice nurse (PN), a tailored care and treatment plan, multidisciplinary care management, and targeted intervention and follow-up over time.

Due to its multi-component nature and integration in the dynamic primary care setting, the [G]OLD home visitation programme can be characterised as a complex intervention. Besides investigating the effects on patient outcomes in a large-scale controlled trial, it is equally important to obtain a profound understanding of how complex interventions function in their intended context [15–17]. Therefore, we prospectively designed a process evaluation to follow the implementation of the [G]OLD home visitation programme from its initial use until continued use [18]. Such pre-planned process evaluations performed alongside the effect evaluation allow for in-depth information to differentiate between interventions that have shortcomings in itself (intervention failure) and those that are badly delivered (implementation failure) [19,20]. Instead of merely implementing, the present home visitation programme required general practices to redesign their care delivery for potentially frail older people from reactive, disease-oriented care towards proactive, patient-oriented care. As a result, the objectives of the process evaluation are to examine (1) the extent to which the [G]OLD home visitation programme was implemented as planned in general practices, and (2) the extent to which general practices successfully redesigned their care delivery.

## Methods

### Process evaluation design

The process evaluation plan was designed according to seven theoretical elements proposed by Saunders and colleagues [21], as adapted from Baranowski and Stables [22] and Linnan and Steckler [23]: fidelity (quality of implementation), dose delivered (completeness), dose received (exposure; satisfaction), reach (participation rate), recruitment, and context. The element ‘context’ was explored into more detail using the Normalisation Process Model [24] to identify factors that affect the success or failure of delivering and implementing the intervention in a dynamic and complex primary care setting. The process evaluation questions per component of the process evaluation are summarised in Table 1. Further details concerning the design of the process evaluation plan are discussed elsewhere [18].

**Table 1:** **Process evaluation questions and data collection tools per component of the process evaluation plan**

**Component**	**Process evaluation questions**	**Data collection tools**
Fidelity (quality of implement-tation)	1) To what extent were all elements of the home visitation programme implemented as planned?	Semi-structured interviews GP/PN; structured registration forms
	2) Is care delivery for older people within general practices successfully redesigned?	
	a. Did GPs and PNs change their mindset from delivering reactive care to proactive care?	
	b. To what extent were linkages established with other professionals or organisations?	
	c. Did general practices receive useful decision-aids to support decision-making?	
	d. Is a registration system realised that is (practically) useful for general practices?	
Dose delivered (completeness)	3) To what extent did PNs follow all steps of the intervention protocol (see Figure 1)?	Semi-structured interviews GP/PN and older people; structured registration forms
Dose received (exposure)	4) To what extent were older people compliant with follow-up actions formulated in the care and treatment plan?	Structured registration forms
Dose received (satisfaction)	5) To what extent were GPs and PNs satisfied with organising care according to the home visitation programme?	Semi-structured interviews GP/PN
	6) To what extent were older people satisfied with the home visit?	Semi-structured interviews older people
	7) To what extent did older people benefit from the home visit and, if necessary, subsequent follow-up actions?	Structured registration forms
Reach (participation rate)	8) What proportion of the intended target population participated?	Registration trial database
	9) What were the reasons for non-participation of older people?	Reminder non-responders and notes in trial database
	10) Was the right target population reached according to GPs/PNs?	Semi-structured interviews GP/PN
	11) What proportion of older people completed all steps of the intervention protocol?	Registration trial database
	12) What were the reasons for drop-out of older people enrolled?	Notes in trial database
Recruitment	13) What procedures were used to recruit general practices and older people for participation?	Narrative report by project team
Context	14) What barriers and facilitators influenced implementation of the home visitation programme within general practices?	Semi-structured interviews GP/PN
	15) To what extent did the control group receive the intervention or similar types of proactive care (contamination)?	Short semi-structured interview GP by phone
*Interactional workability*	16) To what extent was congruence accomplished between PNs and older people and GPs and PNs regarding detected (health) problems and/or follow-up actions?	Semi-structured interviews GP/PN and older people
*Relational integration*	17) Did PNs have sufficient knowledge, expertise and skills to perform the activities as part of the home visitation programme?	Semi-structured interviews GP/PN and older people; evaluation form during training session
	18) To what extent did PNs feel confident that they could assess and address older people’s health problems?	
*Skill-set workability*	19) Was the division of work between GP and PN acceptable?	Semi-structured interviews GP/PN
*Contextual integration*	20) Did the home visitation programme fit within the range of health care services offered by general practices?	Semi-structured interviews GP/PN
	21) Were sufficient resources (e.g., time, staff, and money) available for the adequate performance of the home visitation programme by general practices?	

The Medical Ethical Committee (MEC) of Maastricht University Medical Centre (MUMC+) judged the protocol of the [G]OLD-study and the accompanying process evaluation as not needing formal ethical approval (METC 10-4-015). Nonetheless, the MEC approved the study protocol and related documents. Written informed consent was obtained from participants at recruitment and additional verbal informed consent was obtained for the interviews on behalf of the process evaluation.

### Setting and participants

The process evaluation was conducted parallel to a longitudinal, quasi-experimental trial investigating the effects of the [G]OLD home visitation programme on health-related quality of life and disability [11]. Twenty-four general practices from three regions in the south of the Netherlands (‘Maastricht-Heuvelland’, ‘Parkstad’, and ‘Midden-Limburg’) were involved in the trial and all participated in the accompanying process evaluation. Thirteen general practices were instructed to redesign their care delivery from reactive to proactive care by implementing the [G]OLD preventive home visitation programme between July 2010 and September 2011 (intervention group), whereas 11 general practices offered usual care to older people (i.e., reactive care when older people present themselves with health problems or complaints) (control group). GPs and PNs from the intervention group were the key actors within the home visitation programme and therefore the main sources from which process data were gathered. Mean age of the 14 GPs from the intervention group participating in the process evaluation was 46.8 years (SD = 7.9; range: 31–60), 64.3% was male, and their average working experience as GP was 17.6 years SD = 8.5; range: 4–30). Thirteen PNs (one PN worked in two participating general practices; one general practice had two PNs) were responsible for implementing the home visitation programme. In several countries, including the Netherlands, PNs increasingly substitute the GP in chronic disease management and in care for older people now as well [25–27]. PNs had a mean age of 38.0 years (SD = 10.8; range: 22.6-57.3) and were predominantly female (91.7%). Their mean working experience as PN was 2.6 years (SD = 1.8; range: 0.5-6.5). Finally, the experiences of older people were incorporated. The target population were all community-dwelling older people aged 75 years and older who had been selected by general practices from their GP Information System. Older people not living independently, those on a waiting list for admission to a nursing home or home for older people, those under close medical supervision (chemotherapy, chronic haemodialysis or other therapies posing a high burden on the older person), and the terminally ill were excluded. The remaining older people eligible to participate were referred to as potentially frail older people whose frailty status would be judged by the CGA during a home visit.

### [G]OLD home visitation programme

**Figure 1:**
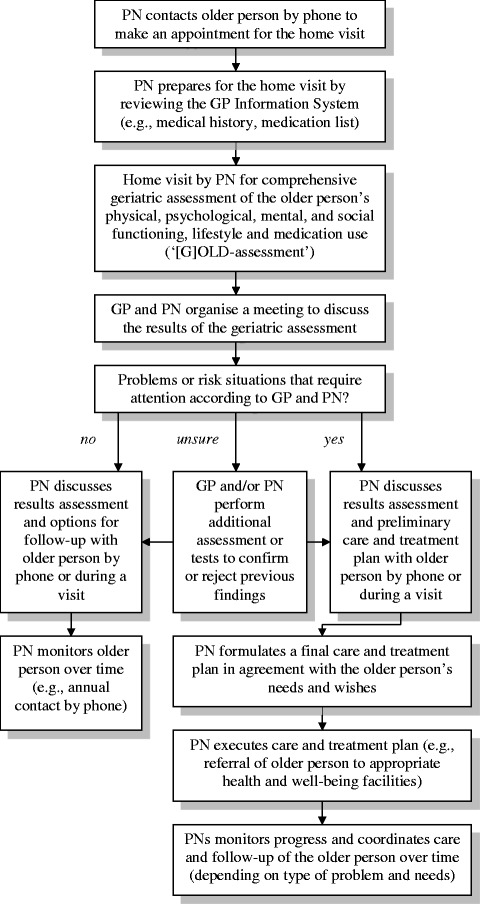
**Steps of the [G]OLD home visitation programme (intervention protocol).**

Figure 1 illustrates the steps of the [G]OLD home visitation programme that need to be undertaken by PNs, in collaboration with the GP, to ensure optimal delivery of the intervention to older people. The active ingredients of the [G]OLD home visitation programme for it to reach the intended effects were a home visit for conducting a CGA, a tailored care and treatment plan, multidisciplinary care management, and targeted intervention and follow-up. Although PNs could adapt certain steps (i.e., how to arrange follow-up of older people over time) to older person’s needs and their own working routine, no steps were allowed to be omitted. PNs used the [G]OLD-instrument, which is a CGA to obtain a complete overview of older people’s physical, psychological, mental, and social functioning, as well as lifestyle and medication use. This instrument was specifically developed for application by PNs in general practices [28]. If required, PNs could administer part two of the [G]OLD-instrument consisting of more elaborate tests concerning cognition, depression, and personality disorders.

Besides these steps, care delivery to older people within general practices could only be successfully redesigned if four additional aspects from the Chronic Care Model [12] were realised. In line with the underlying philosophy of the intervention, general practices had to change their mindset from delivering reactive care towards offering proactive care (‘delivery system design’). In order to achieve this, PNs were required to perform proactive home visits for the early identification of health and well-being problems among older people, followed by the other steps of the [G]OLD home visitation programme as depicted in Figure 1. In addition, to offer individually appropriate care to older people, PNs were instructed to achieve interdisciplinary collaboration by establishing linkages (e.g., multidisciplinary meetings) with (local) professionals or organisations offering care and/or well-being services to older people (‘community resources’). Further, the project team delivered decision-aids and tools to assist general practices in deciding about the presence or absence of problems detected through the CGA, as well as a service map of where to refer older people with specific problems to within the available range of health and well-being services (‘decision support’). Finally, the local primary care organisations were instructed to realise an ICT-based clinical information system within existing systems in general practices to register the findings from the CGA and the results of monitoring and follow-up of older people over time (‘clinical information systems’).

Before the start of the intervention period in July 2010, all PNs participated in a two-day training session that focused on gaining knowledge and skills to carry out the different steps of the home visitation programme. PNs received background information on the [G]OLD-instrument, they practiced applying the [G]OLD-instrument among older people, and they were brought into contact with several professionals or organisations offering care and/or well-being services to older people.

### Data collection

Mixed-methods research was conducted in which quantitative and qualitative data complemented one another to yield an enriched understanding of the implementation process and of the extent to which general practices redesigned their care delivery. Using a convergent-parallel approach, quantitative and qualitative methods were given equal priority, data were gathered concurrently, and integration took place at the interpretation or conclusion stage of the research. This resulted in a so-called parallel-databases design [29] or also called a triangulation design model, which is frequently applied in primary care research [30]. Apart from the use of quotes, the qualitative components of our study adhered to RATS guidelines [31]. An overview of the data collection tools per research question guiding the process evaluation is presented in Table 1.

### Semi-structured interviews

Individual semi-structured interviews were conducted with PNs and GPs in the general practice and with older people in their homes. Semi-structured interviews are well suited to explore people’s opinions and perceptions with flexibility and at the same time cover fixed elements as a part of the process evaluation. The process evaluation questions guided the topic lists for the interviews. All 13 participating PNs were interviewed three times (i.e., after three months, six months, and at the end of the intervention period) to gain a detailed overview of their experiences in various phases of the implementation process. For example, their experiences with conducting the home visits and the extent to which general practices redesigned their care delivery from reactive to proactive care were among the topics being discussed. The interviews lasted approximately 30 to 60 minutes each.

At the end of the intervention period, one GP from each general practice participated in a 30-minute interview. In general practices with several GPs, the one who was most closely involved in implementing the home visitation programme was approached. As a result, 13 interviews were conducted with 14 GPs (in one general practice both GPs participated in the interview). Examples of topics discussed were the involvement of the GP in the home visitation programme, the extent to which a transition from reactive to proactive care was achieved, as well as intended continuation of the home visitation programme over time within the general practice. At the end of the follow-up period of the trial (18 months after baseline), one GP per control practice was interviewed shortly by phone to determine the extent to which the general practice had been involved in proactive care similar to the intervention group (contamination).

After the home visit, older people were invited to share their experiences and views regarding the home visitation programme. Interviews lasted approximately 30 minutes and took place three to five weeks after the home visit. This gave PNs sufficient time to communicate the care and treatment plan to the older person, while the risk that the older person would not remember details of the home visit anymore was not yet too high. Initially, one older person (or a couple, if both received a CGA) per general practice/PN was purposefully selected (total n = 17 older people, including 3 couples) in consultation with the PN based on the principle of maximum variation by taking into account gender, age, household status (living alone vs. living together), and health status (no/few problems detected vs. multiple problems detected). After the initial interview round, data saturation was reached as no new themes or issues arose during the coding process.

### Structured registration forms

Several structured registration forms were distributed among PNs to gain insight into complete and acceptable delivery of steps of the home visitation programme. PNs received the [G]OLD-instrument on paper, which also included a form to register details of the post-discussion with the GP, and the care and treatment plan according to the official format as recommended by the Dutch College of General Practitioners [32]. The care and treatment plan contained details per detected problem of the goal to be achieved, who will undertake action, and when evaluation will take place. Returned forms were checked for completeness and accuracy of reporting by a research assistant, thereby serving as a proxy for complete delivery of elements of the intervention protocol.

At the end of the 18-month follow-up period, PNs were asked to register on a structured form for each older person the number of follow-up contacts, the extent to which this person in general complied with follow-up actions or advice given (5-point scale: ‘always’, ‘most of the time’, ‘sometimes’, ‘rarely’, ‘never’, including the options ‘I don’t know’ and ‘not applicable, no action/help needed’), and the extent to which the older person in general benefitted from follow-up actions, referral or advice given (4-point scale: ‘very much’, ‘somewhat’, ‘a little’, ‘not at all’, including the reason for their judgement).

The two-day training programme for PNs was evaluated using a structured evaluation form to be filled-out at the end of each day. Besides their satisfaction with the training in general (on a scale ranging from 0 (worst score) to 10 (best score)), PNs were asked to what extent the training prepared them for the performance of the home visits and to what extent they felt confident that they could perform a home visit independently (both measured on 7-point scales ranging from 1 (‘not at all’) to 7 (‘very much’)).

At baseline, older people received the [G]OLD care diary to register details of their contacts with professionals offering care and/or well-being services during the 18-month follow-up period. This information was intended as an indicator of compliance to follow-up actions besides the judgement of the PN. Unfortunately, only 7.1% of the older people (n = 42 out of 590 older people who had a home visit) filled-out the [G]OLD care diary and returned it at 18-months follow-up. According to some of the older people who gave remarks on the use of the [G]OLD care diary, they did not see the added value of it and/or forgot to fill it out after contacts with professionals. Since we did not obtain a representative sample for estimating compliance, this information was not used for process evaluation purposes.

### Continuous registration and notes

The number of participants, non-participants, and drop-outs, including reasons for non-participation and drop-out were registered by members of the research team in the trial database. Furthermore, members of the research team made notes or narrative descriptions of the recruitment procedures and feedback meetings with all PNs together. These meetings were organised twice: after approximately six months and at the end of the intervention period. PNs were offered the possibility to exchange experiences and interim results of the individual semi-structured interviews were discussed (member check).

### Data analysis

Descriptive statistics (e.g., means, frequencies, and percentages) were computed for quantitative process data using IBM SPSS Statistics for Windows, version 21.0. All individual semi-structured interviews with GPs, PNs, and older people from the intervention group were digitally recorded, after obtaining verbal consent from participants, and transcribed verbatim. The analysis of the interview transcripts was supported by the software package NVivo 7. Two members of the research team (PhD-student, who also performed all interviews, and a research assistant; both with an academic background) independently coded the data to enhance credibility of the findings. One member of the research team coded all transcripts (PhD-student), while a second coder (research assistant) served as a control and coded a random selection of one quarter of the transcripts to reduce workload. A general inductive approach was applied in which the coding process was guided by a coding tree based on the process evaluation objectives. This relatively simple approach allows for deriving findings in the context of focused evaluation questions [33]. Systematic and rigorous reading and coding of the transcripts allowed major themes to emerge, which were compared for overlap. Consensus was reached after discussion and since the most important themes had already emerged, it was not considered necessary for the second coder to analyse the remaining transcripts. Credibility of the qualitative findings was also enhanced using member checks (i.e., preliminary findings were documented and send to PNs for feedback) and method triangulation (i.e., using multiple methods to collect data on a particular process evaluation question). Other qualitative data (i.e., notes of the feedback sessions with PNs) were analysed using conventional content analysis [34]. Direct information is obtained from participants without imposing preconceived ideas on them, which allows categories or new themes to emerge from the data that did not yet emerge from the semi-structured interviews. Descriptions of procedures applied by the research team for recruitment of general practices during the intervention period were summarised in a narrative report.

## Results

The results of the process evaluation are described below, structured according to the process evaluation questions as presented in Table 1.

### Fidelity (Q1)

All steps of the home visitation programme were largely implemented as planned. Exceptions are PNs who changed or omitted questions in the CGA, no or delayed post-discussion between the GP and PN, and no or inappropriately formulated care and treatment plans. Both PNs and GPs struggled most with how to arrange long-term monitoring of older people considering the limited number of hours PNs could dedicate to care for older people. Besides this, each PN developed his/her own routine in performing the different steps.

#### Change in mindset (Q2a)

The shift from reactively to proactively approaching older people in a structured and comprehensive way was evident for PNs, as well as for most GPs. One PN mentioned that GPs were not used to approaching older people in a proactive way. They usually offer care and/or treatment upon request, whereas PNs are more familiar with delivering preventive care.

At the end of the intervention period, all general practices intended to continue with the home visitation programme, but the proactive versus a more reactive approach posed a dilemma for half of the practices. Proactively visiting all older people (75+) allows for primary prevention of problems but is a huge time investment for general practices. Purposefully visiting older people who show signs of decline would be more feasible, but this is at the expense of detecting problems which could have been prevented if addressed earlier.

#### Interdisciplinary collaboration (Q2b)

Due to the home visitation programme, PNs’ extended their network of professionals or disciplines involved in care for older people and they used their network to a greater extent. The number of referrals to secondary care was limited and the majority of contacts took place within primary care, for instance with home care organisations, physiotherapists, and occupational therapists. Several PNs indicated that they only had few contacts with other care professionals since not that many problems had been detected. Collaboration with other professionals was facilitated when they were located nearby, preferably in the same building.

In three general practices, multidisciplinary meetings took place on a structural base (e.g., once a month) already before the start of this study, and older people were discussed in these meetings from time to time as well. In other general practices, no structural meetings were organised yet with disciplines outside the general practice, mostly because no or only few complex problems were detected among older people. Three general practices had concrete plans to organise multidisciplinary meetings in the near future. Others were not convinced of their added value compared to existing meetings or contacts with other disciplines on an individual base.

#### Decision-aids to support decision-making (Q2c)

Half of the PNs used the service map made available by the project team and considered it useful. Others had not encountered any situations in which the service map could have been helpful.

Both PNs and GPs were positive about the [G]OLD-instrument. Its extensiveness offered a comprehensive overview of the older person’s health and well-being, yet the instrument was time-consuming to administer. Consequently, several PNs and GPs sought for a balance in restricting the time investment without losing important content (e.g., application to a limited group of older people).

#### Clinical information system (Q2d)

Only in the region ‘Parkstad’, the digital system for registering the findings from the CGA and the care and treatment plan was finished at the start of the intervention period, although PNs could not yet register the results of monitoring and follow-up of older people over time. Initially, PNs considered it time-consuming and double work.

### Dose delivered (completeness) (Q3)

**Figure 2:**
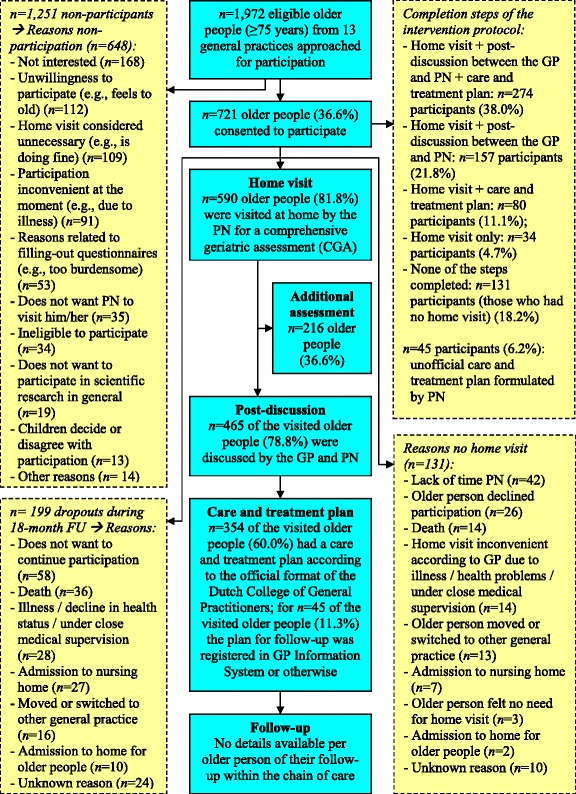
**Dose delivered and reach of the [G]OLD home visitation programme.**

The dose delivered is illustrated in Figure 2. In total, 590 participants were visited at home by the PN for a CGA between July 2010 and September 2011. An underestimation of the actual number of post-discussions is likely, since not all PNs consistently filled-out the registration form. The percentage of formulated care and treatment plans per PN varied widely from 4.0% to 95.2%. In case PNs did not use the official format, we did not count them as care and treatment plans. Finally, we had no valid details per older person of their follow-up within the chain of care as PNs could not easily differentiate between follow-up contacts on behalf of the home visitation programme and other contacts with the general practice within the 18-month period. Nonetheless, the follow-up process might have been suboptimal in several cases, as some PNs experienced time constraints and/or they did not have a concrete plan for monitoring and follow-up (this was not provided as part of the intervention protocol).

### Dose received (exposure) (Q4)

According to the forms filled-out by PNs (*n* = 384, 65.1%), of the 229 older people who received follow-up actions or advice, 67.7% complied ‘always’ or ‘most of the time’, while 10.5% complied ‘rarely’ or ‘never’. Six older people admitted during the interview that they did not comply with a specific advice given by the PN.

Half of the PNs noticed that they often came across older people who did not want any follow-up action(s) in the first place. Especially mental problems were difficult to deal with. Often older people agreed to undertake actions when it was already too late. Some PNs struggled with how to deal with these older people and how to find a balance between respecting the older person’s wishes and maintaining contact to try to achieve the desired actions over time.

### Dose received (satisfaction) (Q5-Q7)

#### Practice nurses (Q5)

PNs liked the performance of the home visits because of the ability to get to know the older person and to offer help or advice. The home visit lowers the threshold for older people to contact the general practice should problems arise in the future. PNs were in favour of a home visit instead of a consultation in the general practice, as it offered a more objective picture of the older person’s functioning. At the general practice level, the home visitation programme resulted in more attention for older people in general and closer collaboration between the GP and PN in organising care for older people. The majority of the PNs did not like the administrative work. Moreover, opinions of PNs diverged regarding the added value of the care and treatment plan over and above the registration of follow-up actions in the GP’s Information System. In general, PNs evaluated the preparatory training rather positively (M = 7.64, SD = 0.50 for the first day vs. M = 6.64. SD = 0.92 for the second day).

#### General practitioners (Q5)

Half of the GPs liked most that they obtained a comprehensive and complete picture of older people’s functioning and the social network surrounding older people. As a result, GPs considered the home visits useful, because it offered them additional information which might be valuable for future reference. At the general practice level, the home visitation programme had offered a starting point and useful tools for organising care for older people. Seven GPs mentioned that, against their expectations, no or only few previously unknown problems were detected. Furthermore, the older people that were out of the picture according to the GP and that would therefore particularly benefit from the home visit often did not consent to participate.

#### Older people (Q6)

Although the majority had no specific expectations about the home visit, three older people were hesitant about the purpose of the home visit at first. All older people were very satisfied with the home visit and afterwards, they had a good feeling about it or they emphasised that it had been interesting. They were very positive about the PN and felt that they could discuss everything with him/her. The home visit was neither too short nor too long and they felt that everything that they considered to be important was discussed. The questions asked as part of the CGA were not difficult to understand, impolite, awkward, or strange. Older people liked the ability to talk about different things, the unexpected attention from their general practice, and the fact that they now had a familiar face in the general practice. One person indicated that these visits tend to go towards too extensive meddling with other people’s affairs, especially among older people who are doing relatively well and do not have a specific request for help.

#### Benefits to older people (Q7)

According to the forms filled-out by PNs (*n* = 394, 66.8%), for 29.9% of the older people follow-up actions, referral or advice had been ‘very much’ or ‘somewhat’ beneficial, while the remaining 70.1% of the older people benefitted ‘a little’ or ‘not at all’. Most PNs indicated that the majority of older people experienced few benefits, because no problems had been detected or only problems that could be addressed easily or problems that had already been taken care of. As recognised by a PN, a GP and confirmed by one older person, sometimes older people did not optimally benefit from the home visit because they to some extent hold a façade of normalcy. Finally, one GP commented that the home visits are less useful for older people without a specific request for help as they often do not want to undertake action.

Six older people mentioned that the home visit had been useful for them, mostly because it lowered a threshold to discuss matters for which they do not easily contact the general practice themselves. Others believed that you are just old and there is nothing that can be done about that, and that certain problems (e.g., loneliness) cannot be solved.

### Reach (Q8-Q12)

Of the 1,972 eligible older people (≥75 years) approached, 36.6% consented to participate (see Figure 2). Mean age of participants was 80.6 years (SD = 4.26; range: 74.4-95.4) and 56.0% (*n* = 972) were female. Participants were significantly younger compared to non-participants (M = 81.2, SD = 4.39) (*p* = 0.004) and men were 1.43 times more likely to participate than women. Participants who dropped out (27.6%, *n* = 199) during the 18-month follow-up period (see Figure 2), received usual care if necessary. Drop-outs were significantly older (M = 81.1, SD = 4.76) compared to those who continued participation (M = 80.3, SD = 3.95), (*p* =0.027) and women were equally likely to drop-out as men (OR = 0.99). As Figure 2 shows, for only 38.0% of the 721 participants all steps of the [G]OLD-protocol up to the follow-up process were completed according to the registration forms filled-out by PNs.

Nearly all PNs and several GPs believed that they had missed the older people who would particularly benefit from the home visitation programme, since participants were the relatively healthy older people, those for whom care was already arranged quite well, or the ones who often visit the practice. They felt that due to the informed consent procedure of the trial, people who are not doing well or the more frail older people are suspicious about the consequences of participation.

### Recruitment (Q13)

All general practices in the regions ‘Maastricht-Heuvelland’ and ‘Parkstad’ were informed about the [G]OLD-project by means of a letter from the primary healthcare organisation of their region, followed by information sessions and practice visits for those interested to participate. Non-responders were contacted by phone to inquire about their willingness to participate in the control group. Since insufficient general practices agreed to participate in the control group, jeopardising the continuation of the trial, the recruitment of control practices was extended to another region (‘Midden-Limburg’). In total, 188 general practices from three regions were approached for participation and 24 general practices consented to participate (12.8%). Thirteen general practices were included in the intervention group (7 from the region ‘Parkstad’ and 6 from the region ‘Maastricht-Heuvelland’) and 11 general practices in the control group (2 from the region ‘Parkstad’ and 9 from the region ‘Midden-Limburg’).

Older people were approached for participation by means of an information letter and consent form. In the intervention group, those who did not return the signed consent form within two weeks were contacted by phone once to inquire whether they received the information letter. One postal reminder was send to older people who could not be contacted by phone. Due to the substantial time investment of calling older people, non-responders in the control group only received a postal reminder.

### Context (Q14-Q21)

#### Barriers and facilitators for implementation (Q14)

Most of the barriers experienced by PNs during implementation were related to logistical difficulties in planning the different steps of the home visitation programme alongside other daily work. Especially the introduction of a new disease management programme for cardiovascular risk management (CVRM) by the primary care organisations during the intervention period posed challenges to PNs and GPs, causing several PNs to invest less time in the home visitation programme than planned. Finally, barriers for continuing the home visitation programme over time were the lack of an adequate reimbursement by health insurers of the costs of care for older people and the overall time investment of the home visitation programme (total time investment from preparation of the home visit to formulating the care and treatment plan was on average 85 minutes per older person).

A facilitator for implementation according to several PNs was gaining routine in efficiently planning and executing the different steps of the home visitation programme. Moreover, some PNs expressed the need for regular meetings with other PNs to exchange experiences or the ability to consult an expert panel with practical questions. For GPs, having a PN in the general practice who is largely responsible for performing the home visitation programme and who gained experience in it, was a positive development conducive to successful implementation.

#### Contamination control group (Q15)

None of the participating general practices in the control group had been involved in any form of proactive care for community-dwelling older people during the 18-month study period.

#### Interactional workability (Q16)

All PNs were satisfied with how they worked together with the GP in deciding about follow-up actions for detected problems. Yet one PN and one GP noticed that sometimes there was incongruence between the two of them: the proactive approach required GPs to address different kinds of problems and/or needs that otherwise might not have received attention at that point in time. Mostly, older people agreed with the suggestions done by the PN for follow-up actions. However, both PNs and GPs observed that quite a few older people were not willing to undertake any follow-up actions or only when problems had progressed substantially (incongruence).

#### Relational integration (Q17 + Q18)

At the end of the training programme, PNs felt very confident that they could perform a home visit independently (M = 6.27, SD = 0.91). In general, both PNs and GPs thought that the PN’s knowledge, expertise or skills regarding care for older people were sufficient and, according to PNs, even increased during the intervention period. Those inexperienced with structural assessments would benefit from feedback, supplementary information, or examples on how to administer certain tests of the CGA and how to assign a score to the answers given by older people. A few PNs did not have that much expertise yet in deciding whether or not to undertake follow-up actions for detected problems and in formulating care and treatment plans correctly. One GP sensed a lack of affinity of the PN regarding care for older people. All older people believed the PN had sufficient knowledge about health, listened to them, took sufficient time for the home visit, and respected their needs and wishes.

#### Skill-set workability (Q19)

Both PNs and GPs considered the division of work regarding the home visitation programme clear and acceptable. As GPs often cannot attribute as much time to older people as they would like, the expertise gained by PNs in care for older people was very much appreciated. One PN was a little disappointed that the GPs did not use her expertise more often in arranging follow-up actions for older people.

#### Contextual integration (Q20 + Q21)

Six PNs felt that the home visitation programme was well integrated within the health care services offered by the general practice. According to two GPs, the home visitation programme fits within the health care services offered by general practices, as older people are familiar with the general practice. Furthermore, it enabled GPs to be the central care provider and to collect information that is relevant to them.

According to PNs and GPs, sufficient time for care for older people was an essential resource for adequate performance of the home visitation programme. PNs’ available time had to be carefully divided over various patient categories within the general practice. Five GPs thought that the time investment was disproportionate compared to the benefits in terms of detected problems. The other GPs thought the time investment was justifiable, as it yielded a lot more information about the older person.

Opinions of GPs diverged with respect to the importance of the reimbursement policy of health insurers for implementing care for (frail) older people. While some GPs considered it to be of minor importance, others believed its importance will grow over time due to competition between various disease management programmes for the available time of the PN. Finally, some GPs stated that continuation of the home visitation programme would largely depend on it.

## Discussion

This paper reports on a pre-planned, theory-based process evaluation into redesigning care delivery by general practices and implementation of a home visitation programme for potentially frail community-dwelling older people (aged ≥75 years). The process evaluation plan was structured using the theoretical elements ‘fidelity’ (quality of implementation), ‘dose delivered’ (completeness), ‘dose received’ (exposure; satisfaction), ‘reach’ (participation rate), ‘recruitment’, and ‘context’ as proposed by Saunders and colleagues [19], as well as the Normalisation Process Model [22] to explore the element ‘context’ in greater detail. Overall, the home visitation programme was delivered completely according to protocol to only 38.0% of the 721 study participants. This is considerably lower than the completion rate of 78% of a similar intervention [35]. Several threats to complete delivery of the intervention have been identified.

First of all, lack of time emerged as a crucial factor in various elements of the process evaluation and prior research showed that it is an important barrier in providing structured care to older people in general practice [36,37]. Administering the [G]OLD-instrument during the home visit, post-discussion between the PN and GP, administrative work (e.g., registering the findings from the CGA in the digital registration system), and monitoring older people were (initially) judged to be time-consuming activities or were influenced by time constraints of either the PN or GP. The time investment and corresponding available financial reimbursement were considered disproportionate compared to the benefits in terms of detected problems, thereby influencing the intention of general practices to continue using the home visitation programme. Furthermore, the time investment posed logistical challenges for some general practices in dividing the limited time of the PN over various patient categories. Interestingly, solutions to reduce the time investment were not sought in shortening the [G]OLD-instrument or adapting the intervention protocol in general, but in targeting the home visitation programme to a selected group of older people who benefit most from it.

Secondly, few new or complex health and/or well-being problems had been detected, which was the main cause of no post-discussions and no care and treatment plans for specific cases, and limited interdisciplinary cooperation. Both PNs and GPs believed that the relatively healthy older people were visited, suggesting inadequate reach. On the one hand, volunteer bias may have been introduced by the recruitment procedure on behalf of the parallel quasi-experimental trial. Alternatively, the population-based screening approach may have resulted in few (newly) detected problems among older people. While some opt for population-based screening of older people in general practice [6], other studies suggest that general practices benefit most from a more targeted screening approach [38,39].

While coding the transcripts of the semi-structured interviews, we retrieved relevant information regarding fidelity that went beyond mere quality of implementation. In agreement with Hasson and colleagues [40], we found that implementation fidelity was influenced by the care professionals’ commitment to the home visitation programme, as well as their ability to execute the intervention protocol with the resources at hand. Both PNs and GPs were satisfied with the home visitation programme. It had resulted in more attention for older people, closer collaboration between the GP and PN, and a comprehensive picture of older people’s functioning and social network. This positive attitude made them willing to find solutions for barriers encountered during the implementation (e.g., monitoring older people via other disease management programmes). Such small changes to the intervention protocol were not considered threats to fidelity, but as necessary for translation of the home visitation programme into daily practice (i.e., work patterns of PNs) [41]. However, PNs struggled a lot with the follow-up process and thus, the extent of arranging follow-up actions and monitoring older people over time might have been limited, as also found in another recent study [27], threatening fidelity of implementation. Besides time constraints, other causes may be inadequate guidelines or decision-aids for PNs to arrange the monitoring process, some older people did not want to undertake follow-up actions, and a lack of adequate ICT-support to facilitate registration of the follow-up process.

With respect to redesigning care delivery for older people, shifting of care from the GP to the PN was evaluated positively and appeared to be conducive to the delivery of proactive care, as PNs in general are more familiar with a preventive approach. Nevertheless, some GPs were still more inclined towards offering reactive care. This implies that over time, GPs may relapse into their usual way of delivering care upon request (because of time constraints, lack of benefits in terms of detected problems, etc.), stimulated by the disproportionate cost-benefit ratio of the home visitation programme as mentioned earlier and the predominantly reactive healthcare system in the Netherlands .

All older people were satisfied about the home visit, regardless of whether problems had been detected, mostly because it offered them the ability to express their daily concerns. This ‘attention’ aspect of the home visit has been recognised before and is considered insufficient for eliciting substantial effects on patient outcomes [42].

The development of a prospective process evaluation plan, underpinned by a theoretical framework, and using a mixed-method approach to data collection allowed for a high-quality and more thoroughly conducted process evaluation. Trustworthiness or credibility of the findings was enhanced using, among others, member checks, method triangulation (i.e., using multiple methods to collect data on a process evaluation question), and two independent coders during qualitative data-analysis. Nevertheless, the subjective experiences of PNs, GPs, and older people may have been subject to social-desirability bias or recall bias. Opinions of GPs might have been biased due to negotiations between insurers and care groups for a bundled payment system [43] for complex care for older people. Honest responding by PNs may have been promoted by the independent role of the researcher and relationship of trust created due to repeated contacts during the intervention period. Further, despite using a varied sample of older people, we could not entirely circumvent that older people tend to provide less detailed descriptions of their experiences [44]. Another limitation is that the structured registration forms were not always completely and accurately filled-out by PNs. This made them a less reliable source to assess for example implementation fidelity or dose delivered, and thereby also restricting method triangulation for certain process evaluation questions.

## Conclusions

The current process evaluation offers useful insights for interpreting the results of the parallel quasi-experimental trial and for sustainability of the home visitation programme in general practices [45]. The largest threats to positive outcomes at the patient level are the low dose delivered and inadequate reach. According to PNs, beneficial effects of the home visitation programme were ‘little’ or ‘not at all’ present in the majority of visited older people (70.1%). Despite this, the home visitation programme was judged positively by both GPs and PNs and resulted in positive developments within the general practices. This suggests that the intervention does not have major shortcomings in itself, but the delivery offers room for improvement. Besides selecting the more frail community-dwelling older people with multiple and complex problems, alternative time-saving solutions for general practices may be sought in connecting general practices with initiatives in the neighbourhood or at the community level by developing welfare and care models. Regardless, the involvement of general practices is advocated [4,5] and GPs also believe the home visitation programme fits within their range of care services. Finally, PNs would benefit from on-going training to update their knowledge and skills, thereby enhancing implementation fidelity, and to allow for exchanging experiences with other PNs. An imbalance between the time investment and available financial reimbursement in proportion to the number of meaningful problems detected among older people requires attention to enable continuation of the home visitation programme over time. The development of future complex interventions in the primary care setting should take into account that a pre-planned, theory-based process evaluation alongside the effect evaluation is inevitable to provide in-depth insight into the actual performance of the intervention in the intended context.

## Abbreviations

CGA: Comprehensive Geriatric Assessment
[G]OLD: Getting OLD the healthy way
GP: General practitioner
PN: Practice nurse
